# Development and pilot evaluation of the Children’s Masking Questionnaire

**DOI:** 10.3389/fpsyt.2026.1893936

**Published:** 2026-07-23

**Authors:** Lema Delara, Najette Ayadi O’Donnell, Saffron Baldoza, Hannah Belcher, Jo Halsall, Sarah O’Brien, Lucy Sanctuary, Laura Hull

**Affiliations:** 1 University College London Hospitals NHS Foundation Trust, London, United Kingdom; 2 Masking Research Collective, Bristol, United Kingdom; 3 Centre for Academic Mental Health, Department of Population Health Sciences, Bristol Medical School, University of Bristol, Bristol, United Kingdom

**Keywords:** autism, camouflaging, children, masking, measure development

## Abstract

**Introduction:**

No tools currently exist to measure children’s experience of masking/camouflaging autistic characteristics.

**Method:**

Using co-production approaches, we developed the Children’s Masking Questionnaire (CMQ) and evaluated its acceptability and appropriateness as determined by family members, young people, and professionals through two qualitative studies.

**Results:**

Participants felt the CMQ was useful and appropriate, and gave specific suggestions for improvement. Following refinement, a two-part CMQ (Adult Response and Child Response) and a Good Practice Guide were finalised, and are freely available to be used in research and practice.

**Discussion:**

Future research should evaluate the CMQ in larger samples and compare its appropriateness across different neurodivergent and mental health conditions.

## 1 Introduction

In recent years there has been increasing research, clinical, and public interest into the phenomenon of masking or camouflaging in autism (1, 2). A recent review noted the inconsistency with which research in this area uses and defines terms (3); for clarity, in the present article, we define autistic masking as “concealing or changing autistic traits”. This could be considered synonymous with “camouflaging”, which has been used by some of the authors in previous work (4, 5); however, “masking” was considered the preferred term by the majority of members of the research collective conducting this work. In this article, we specifically focus on masking *of autistic traits*, although we acknowledge the limited extent to which masking of different aspects of identity can be disentangled (6, 7).

Despite the recent proliferation of research into masking, validated tools to measure masking are still somewhat limited (8). The most commonly used tool, the Camouflaging Autistic Traits Questionnaire (CAT-Q; 9), is a self-report questionnaire for adults. Although the CAT-Q has been used with adolescents (10, 11), its appropriateness for younger children is questioned (12, 13). Masking has been measured in autistic children and adolescents through observation (14) and through discrepancy measures, which compare autistic presentation either between different informants (15–17), or between internal and external expressions (18, 19). However, we are not aware of any self-report or informant-report tools to measure masking in autistic children.

One reason for this may be that there is relatively limited previous research into masking in childhood, before adolescence. The vast majority of research has taken place within adult populations (1); although some studies involved asking autistic adults about their experiences in childhood, it cannot be assumed that adult experiences can generalise to childhood (20, 21). Research into masking in autistic adolescents suggests that there are some similarities in masking experiences between adolescents and adults, such as feeling drained and tired after masking (1). However, qualitative research suggests that the relationship between masking and mental health may be more complex for autistic adolescents (6), and that it may be hard to distinguish between masking of autism and that of other characteristics, such as ADHD (22, 23).

Research into masking in autistic children may have been limited due to concerns that autistic children are not able to identify or describe masking, if they do indeed mask. Emerging research in this area provides some evidence that this is possible, although with caveats. In a qualitative study using an adapted photo-elicitation approach, Howe and colleagues (24) explored the concept of masking with autistic children and adolescents aged 10–14 years. They found that using photographs, and sometimes drawings, to facilitate communication helped children to express sometimes complex ideas such as internal conflict when masking. However, some children found it hard to answer open questions about masking, even when supported by parents; when asked to talk about camouflaging, one child said “Yes, off the top of my head I don’t know, but I, I do know that there are things I do.” (24; page 11).

Taken together, the limited qualitative evidence so far suggests that some autistic children do experience masking of their autistic characteristics, and that some of these children are able to report on their experiences. To better understand these children’s experiences, a tool to measure masking in autistic children would be useful. Such a tool might also – with careful use and following thorough evaluation – be useful in educational and clinical settings to help professionals supporting autistic children. Recent commentaries have noted limitations to any single tool or method of measuring masking, due to each autistic person having a different experience, perception, and awareness of masking, as well as individual differences in the communication and self-understanding needed to reflect on masking experiences (3, 12). However within this piece of work we seek to capture some (though not all) behavioural strategies that may be used by some autistic children, and recommend that this approach is complimented through the use of other methods in the future.

### 1.1 The masking research collective

The present article is one output from a co-produced project exploring masking in autistic children and young people. Funded by a fellowship awarded to the last author, a research collective (the ‘Masking Research Collective’) was convened with the aim of understanding and measuring masking in children, following participatory research practices for working with autistic (young) people (25, 26). Members of the collective include autistic young people, parents of autistic children, and autistic and non-autistic researchers, healthcare and education professionals. The broader project, as well as some of the research presented here, was developed and designed collectively, and the findings were interpreted and discussed collectively. We convened, facilitated, and took part in the collective following recommendations for good participatory research practice, including the AASPIRE guidelines (26). For instance, we aimed for power-sharing strategies such as having a rotating chairperson at each meeting. Limitations to participatory efforts were also acknowledged from the start, such as the last author having already obtained funding for the project meaning that the timelines, broad goals, and researcher role were already pre-determined. One aim agreed by the collective was to develop a tool to measure masking that could be used by young people, families, and healthcare and educational professionals, to better understand children’s experiences of masking. In this paper, we present qualitative findings from the initial piloting evaluation of a novel measure of masking in autistic children.

## 2 Materials and methods

### 2.1 Measure development

The format of the measure was agreed through discussion by members of the Masking Research Collective. We agreed that separate sections for children and adults to complete would be useful to capture 1) children’s own experiences which they may not have shared with adults, and 2) the perspectives of adults who know the child well and may observe behaviours that the child is not aware of or able to articulate. We also agreed that a combination of closed and open questions (i.e. listing specific masking behaviours as well as allowing participants to give their own answers) would allow maximum flexibility for responses. Closed questions were included to give prompts for respondents who may not know what masking is, or who may not have identified certain behaviours as masking; open questions were included so that respondents could report additional masking behaviours and add more detail to responses.

Masking behaviours to be included in the closed question section were identified through two processes. Firstly, an informal review of qualitative research into masking in children was carried out, and examples of specific masking behaviours (or types of behaviours) described in these studies were collected.

Secondly, interviews with three autistic children and their parents were conducted to explore how children and their parents talk about masking. Participants were recruited through local autism support groups in the Bristol area of the UK, and ethical approval was obtained from the University of Bristol Faculty of Health Science Research Ethics Committee (approval number 12262). Characteristics and demographics of participants are described in **Table 1**. A photo-elicitation prompt (similar to that used by 24) was used to encourage reflection on masking; children were asked to take photographs related to “times that you feel good about yourself” and “times that you want to hide or change things about yourself”. These photographs were used as prompts for the interview, asking children to reflect on why they took each photograph and what they want people to know about themselves from the photograph. Interviews were unstructured, with parents encouraged to be present for the interview and contribute anything they wished, however the focus was on the child explaining their photographs. Interviews were audio recorded and transcribed, and the transcription was analysed for any mention of specific masking behaviours.

**Table 1:** Characteristics of the families recruited to generate masking behaviour examples.

Family number	Child age	Child age at autism diagnosis	Child gender	Child ethnicity	Co-occurring conditions	Parent completing interview
Family 1	9	7	Female	White British	Hypermobility	Mother
Family 2	11	11	Female	White British	ADHD, Dyslexia	Mother
Family 3	10	4	Male	White British	Gastro-intestinal problems	Mother

N.B. ADHD, Attention Deficit/Hyperactivity Disorder.

Masking behaviours identified through both these processes were listed, and together the Masking Research Collective refined the behaviours to remove duplicates and clarify wording. A first (pilot) version of the questionnaire was developed together, and named the Children’s Masking Questionnaire as ‘Masking’ was identified as a term which is not necessarily autism-specific. The Collective agreed that, although the conceptual focus of this tool is on masking behaviours used by autistic children, it is important that the tool can be used by and on behalf of non-autistic children, including those with other neurodevelopmental conditions and typically developing children. This was considered particularly important as the tool may be used for children and families who are awaiting an autism assessment, and so responses should not be contingent on having a diagnosis of autism.

### 2.2 Children’s Masking Questionnaire (pilot version)

The pilot version of the Children’s Masking Questionnaire (CMQ) was developed with two sections, one for adults to complete and one for children to complete. It was agreed that any adult who knew the child well could complete the adult-response section, including parents, other family members, teachers, or clinicians who worked closely with the child.

#### 2.2.1 Adult response section

The adult response section contained 18 closed questions for adults to answer about a child, where each question described a general masking behaviour and 1–2 examples of that behaviour were also given. For instance: “Around other people, does the child ever change their voice or language used? This might look like (but doesn’t have to include) adjusting speech volume or tone to sound more like other children; using a different vocabulary with other people than they would normally do.” Response options were Never, Rarely, Sometimes, Often, or Always, and there was also an open section for adults to add more detail or examples of specific behaviours next to their response for each closed question. Adults were instructed to answer based on how often this behaviour occurred during the past month, and to record any behaviours they had seen the child doing, or the child had told them about. At the end of the closed questions, adults were asked to add anything else that they think might be relevant in an open question format.

#### 2.2.2 Child response section

The child response section contained 15 closed questions for children to answer, for instance: “Do you ever change the way you speak to fit in with other people?”. Response options were Never, Rarely, Sometimes, Often, or Always, and these were illustrated with a slider where ‘Never’ was represented by one brick, and ‘Always’ by five bricks stacked on top of each other. At the end of the closed questions, children were asked to “Please add anything else you do like this that we didn’t ask about” in an open question format.

### 2.3 Qualitative pilot evaluation

To qualitatively evaluate the first version of the CMQ, two separate studies were conducted at the same time. The first was an online pilot evaluation with families of autistic children aged 8-12, as well as healthcare and educational professionals. The second was an in-person study conducted at a specialist tertiary service for young people, based within a major UK hospital. The aim of these pilot studies was to evaluate the phrasing, formatting, and response options of the CMQ for children, families, and professionals to improve accessibility and appropriateness.

The methods for each study are presented separately below; results and discussion are presented together as all feedback was combined in order to evaluate and refine the CMQ.

### 2.4 Study 1

In Study 1, family members of autistic young people and professionals working with autistic young people were recruited for an online study to pilot test and evaluate the CMQ. Participants were requested to complete an online survey regarding their experience using the CMQ, and were then invited to complete an online cognitive interview with a researcher to go through the CMQ in more detail.

#### 2.4.1 Methods

Ethical approval for the study was obtained from the University of Bristol Faculty of Health Science Research Ethics Committee (approval number 15564). Data collection took place between January and March 2024. All data collection was conducted by the last author.

#### 2.4.2 Participants

Potential participants for the study were contacted through the researchers’ networks and local family support groups in the Bristol, UK area. Participants were eligible if they were a close family member of an autistic child, or a teacher or healthcare professional who regularly worked with an autistic child. Eleven family members (including mothers, fathers, and grandparents), two teachers, and one speech & language therapist (SLT) consented to take part in the online survey. Three family members and one teacher consented to take part in the cognitive interview afterwards. Characteristics of the autistic children being reported on are included in **Table 2**; no demographic information regarding the informants (participants in this study) was collected. Note that some family members reported on the same child, therefore the number of participants is higher than the number of children reported upon.

**Table 2:** Characteristics of the autistic children reported upon in study 1.

Demographics	Autistic children (N = 11)
Age	Mean = 9.3 years (range = 7-14)
Gender	Male (10), Female (1), Other (1)
Ethnicity	White British (10), Jewish (1), Mixed (1)
Co-occurring conditions	ARFID (1), Anxiety (2), ADHD (9), Epilepsy (1), Dyspraxia (4)

N.B. ARFID, Avoidant/Restrictive Food Intake Disorder; ADHD, Attention Deficit/Hyperactivity Disorder.

#### 2.4.3 Procedure

Participants were emailed a link to an online survey which contained a pdf version of the CMQ and a series of questions about the CMQ (Supplementary Material 1). Participants were asked to complete the adult response section of the CMQ themselves, and to read the child response section and consider whether it would be appropriate for the autistic child they knew to complete. If so, participants were asked to complete the child response section of the CMQ with the child, and to add their thoughts on the child’s experience of this to the online survey. They then completed the additional survey questions. Participants were compensated with a £15 voucher as thanks for their time.

Once the survey was complete, participants were asked to contact the researchers if they were interested in completing a follow-up cognitive interview; four participants agreed to this. In the online interview, a researcher shared a copy of the CMQ with the participant, and asked them to use a think-aloud method while reading through the CMQ, followed by prompts about specific aspects of the measure’s design and completion (Supplementary Material 2). Interviews were audio recorded and transcribed. Participants were compensated with a £25 voucher.

### 2.5 Study 2

Study 2 was conducted as part of a broader undergraduate dissertation project, exploring experiences of masking by adolescents with complex conditions. As part of this project, the CMQ was evaluated as a potential tool to be used during initial clinical consultations for these children. Families (young people and adults) attending the service for consultations were asked for their feedback on the appropriateness of the CMQ, as were clinicians working within the service.

#### 2.5.1 Methods

This study was classified as service evaluation, therefore no ethical approval was required according to the UK Health Research Authority (27). We used the HRA decision-making tool to determine that the planned work aimed to provide information on whether a tool such as the CMQ would be appropriate to use as part of current service in that specific setting; there was no comparison between different provisions of service, and both care professionals and service users were asked to consider the appropriateness of the tool. As such it met criteria for service evaluation rather than research. However, all participants were still required to give verbal or written consent (or assent for those aged under 18) before answering the questions, and were reminded that they did not need to answer any question, or give any personal data such as ethnicity or gender identity, if they did not wish to. All data collection was conducted by the first author (LD). Data collection took place between January – March 2024. Data were collected in paper format anonymously (no record of names or identifiable healthcare information), and all responses were typed up and collated so that no quotations were identifiable to a specific respondent.

#### 2.5.2 Participants

Recruitment for this study took place within a specialised service for young people with suspected or diagnosed complex health conditions, including myalgic encephalomyelitis or chronic fatigue syndrome (ME/CFS), based within a hospital in London, UK. The service comprises a multidisciplinary team, including doctors, nurses, physiotherapists, occupational therapists, psychologists, dieticians and social workers. Young people seen by the service are seen within outpatient, inpatient and day settings depending on need.

Clinicians working within the service were sent an email describing the study with a link to participate. Eight clinicians started the study; only three submitted full responses and so these three participants were considered to have completed the study. All three identified themselves as consultant paediatricians. No demographic information on clinician participants was collected.

Young people and any family members attending the service with them were invited to participate. Participants were eligible if the young person was an outpatient at the specialist service; there was no requirement to have an autism diagnosis or to be awaiting autism assessment. Families were approached while they were waiting for appointments in the service waiting area. LD introduced herself and explained the purpose of the study (to get their feedback on a new measure which might be introduced to the service in the future), then asked for consent to conduct the study with them.

15 child-and-parent dyads (15 young people, 15 parents) agreed to take part in the study. Demographic characteristics of the children, and which parent completed the study with them, are reported in **Table 3**. There was no record of whether young people had a diagnosis of autism, or other neurodevelopmental conditions.

**Table 3:** Characteristics of participants in study 2.

Dyad	Child age	Child gender	Child ethnicity	Child first language	Parent
1	12	Female	Arab	Arabic	Mother
2	13	Male	White British	English	Mother
3	11	Female	Black	English	Mother
4	16	Female	Mixed	English	Mother
5	14	Non-binary	White British	English	Mother
6	15	Non-binary	White British	English	Mother
7	15	Female	White British	English	Mother
8	16	Female	White & Asian	English	Father
9	12	Female	White British	English	Father
10	16	Male	Indian	English	Mother
11	12	Female	Black	English	Mother
12	14	Female	White British	English	Mother
13	16	Male	White	English	Mother
14	17	Other	White British	English	Mother
15	12	Male	White British	English	Father

#### 2.5.3 Procedure

Clinicians were emailed an invitation to join the study, including a copy of the CMQ and a link to an online survey. They were asked to read the CMQ and then complete the survey, which included a variety of questions regarding experiences of masking and asking for feedback on the CMQ. For the purposes of this study, only qualitative responses to the open questions regarding the CMQ (Supplementary Material 3) will be analysed.

Families were given a paper copy of the CMQ to read through. LD sat with them in a private space and verbally read out each item in the child and adult response sections, noting down the responses of the child and/or adult accordingly. Any clarifying questions or statements from participants were also recorded, and these qualitative responses will be analysed for the purpose of this study.

### 2.6 Analysis

Qualitative responses from participants in both studies were analysed in the following ways: Cognitive interviews from Study 1 were transcribed by LH (using automated AI transcription features from Microsoft Teams, which were then checked and corrected by the researcher). All survey and interview responses from Study 1 were read by LH, and key feedback for each CMQ question was summarised by LH as well as feedback for the CMQ as a whole. Qualitative responses to questions from Study 2 about the CMQ as a whole were summarised by LD. These summaries were then shared with the Masking Research Collective for discussion and refinement of the CMQ. The revised Children’s Masking Questionnaire is presented as Supplementary Material 4-6.

## 3 Results

### 3.1 Feedback regarding individual questions

Participants, particularly those completing the cognitive interviews, gave detailed feedback on the wording and content of some questions. This included recommendations on how to make questions clearer to interpret (e.g. adjusting a question which included a double-negative), and using more “straightforward” language (“when you say ‘suppressing’, that might be open to interpretation … would everybody who answers understand that?”). Much of the feedback around specific questions was positive, with participants noting how “clear and simple” the language was.

Many participants expressed appreciation for the inclusion of specific masking behaviours that they felt were common, but had never been identified by professionals before, such as pretending to understand a social situation. For instance, one teacher said “I definitely have seen that in an autistic child in the class, like if children are messing around or shouting silly things, he will laugh when I don’t think he actually understands quite what they’re doing”.

Some adult participants suggested that the children/young people they knew might find it hard to answer specific questions about certain masking behaviours. For instance, one parent noted that their child was not aware they made repetitive sounds, and so would not be able to answer a question about masking those sounds. Another said, “my child says she wouldn’t want to be asked some of these questions because they sound ‘weird’, like flapping your hands when excited”, suggesting that if children don’t use a particular masking strategy, they might disengage from the other questions. However, there were no questions where more than one participant felt that the content of the question (i.e. the masking behaviour being assessed) was not relevant to them. Participants also acknowledged that, even if they weren’t directly familiar with the types of masking described in some questions, they recognised them from other neurodivergent people or families: “I mean he doesn’t really do that, but there was a lot of it that I’ve heard from other parents, these are things that their children often do”. We therefore decided not to remove any questions, to best capture a wide range of masking behaviours, with the understanding that not all questions may be completely relevant for all individuals completing the CMQ.

### 3.2 Feedback regarding the CMQ as a whole

#### 3.2.1 Structure of the CMQ

Participants gave extensive feedback on the structure of the CMQ. As a reminder, this initial version of the CMQ comprised an adult-report section and a child-report section, with both containing closed questions asking about specific masking behaviours as well as open questions asking for more detail on those masking behaviours, or on other behaviours not asked about.

The response formats, particularly the child-response options (using stacking bricks to represent greater frequency of behaviour) were seen as helpful. One participant noted “it’s hard to differentiate between ‘rarely’ and ‘sometimes’, so being able to see the difference between them was useful”. However, some members of the Collective felt that using bricks, which could be stacked infinitely, made it hard to identify what the ‘end point’ was – was three bricks the largest amount possible, or five? A suggestion was made to instead use the image of a battery or loading bar (see Supplementary Material 5), where it would be clear what “Always”, “Sometimes” or “Never” represented out of a total possible amount. One professional, and one parent, also suggested that having tick-boxes would make it quicker and easier to complete, although the Collective were concerned that this might discourage respondents from completing the open questions, as well as lose nuance around the frequency of certain masking behaviours.

Some participants – particularly parents or grandparents of autistic children – noted that they did not try completing the Child Report section with their child/young person, because they assumed they would not be able to complete it. One parent said “He’d be able to read it, but he’d get distracted by the question and not really take in what it meant”. Another said “They are quite hard questions for a child to answer”. However, some participants reported that their child surprised them by engaging with the questionnaire: “I didn’t think he would have done it, but he asked me what I was doing and … he was happy filling it out himself”. Responses varied depending on the child/young person’s age, reading abilities, and neurodivergence, suggesting that flexibility is needed in how the Child Report section is administered. To encourage that flexibility, the Collective decided to move the open questions to the beginning of the Child Report section (see Supplementary Material 5), to allow children to express their own perspective on their masking first, before being prompted with more specific closed questions regarding specific masking behaviours.

A common piece of feedback regarding the Child Report section was that children’s answers varied depending on the situation in which they were answering – specifically between school and at home. One parent said that their child struggled to answer questions because the range of possible situations to mask in was too broad: “you should make it quite direct, you could say ‘when you’re with your friends’, or at school or at home”. Similarly, one teacher noted that “we don’t know how they’re like at home and they may put on an act at school”. In response to this, the Collective agreed that giving children the option to say whether they use each masking strategy at home, at school, or in other places, would allow for more nuance in understanding children and young people’s experiences of masking.

#### 3.2.2 Appropriateness of the CMQ

One key question that the researchers wanted to address was whether the Child Report section is considered appropriate for children and young people to complete. Responses from participants generally indicated that they felt the Child Report section was useful, when children/young people were able to complete it: “It’s really important to try and get the child’s voice into the assessment and find out, what are you actually doing?”. Participants also noted that children may have a different perspective to adults on their masking behaviours, and having both “could be really valuable”. One teacher noted that children may not be aware that they are masking, and so the Child Report section may not be informative: “It’s fine to ask them, so they feel like they’re included, but I don’t feel like it gives a very true picture. I don’t think it’s a conscious thing, really”.

A concern raised by some participants, which was not anticipated by the Collective, was that talking about masking with younger children might encourage them to mask more, or bring up emotional challenges that they were not equipped to deal with (“I didn’t want to trigger them”). Other participants expressed concerns around talking about feeling different if children or young people didn’t feel that way already. One parent said “if they don’t even know they’re autistic yet, this is new to them, and they didn’t even need to know…. I don’t want to make them worry about themselves”. However other participants – especially those who were neurodivergent themselves – talked about the “reassurance” that came from discussing masking experiences with other people: “this is exactly her experience, like she’s realised that she masked. And then also myself … this is exactly what I’m doing, you’re explaining what I’ve been hiding”.

Another common piece of feedback was uncertainty around what to do with the CMQ answers once it had been completed. This was particularly the case for professionals who were considering how they might use the CMQ within their regular practice: “it brings up the issue of what to do with the results”. Similarly, parents wanted to understand how to interpret the answers, ideally in collaboration with professionals: “You should add a section for parent, child and doctor feedback so we can see what the next steps are”. Although providing recommendations on clinical or educational interventions is beyond the scope of the current project (and there is very little research exploring how best to support young people who are masking), the Collective also felt it was important that guidance was provided to help interpret CMQ answers. As a result, we co-developed a Good Practice Guide (Supplementary Material 6) which provides information on how to complete the CMQ and how to use the answers at an individual level. This resource encourages reviewing all the answers to produce a summary of the individual’s masking experiences, and comparing across the perspectives of the adult informant and the young person/child themselves. It also offers advice for families, and for educational and healthcare professionals, on the next steps to take when working with the individual to help them understand their own masking and the impact it has on them.

## 4 Discussion

Through two studies we performed a pilot evaluation of the Children’s Masking Questionnaire (CMQ). On the whole, participants responded positively to the CMQ, and many expressed appreciation for a tool which could help children, young people, and their families better understand their masking. We made specific changes in response to feedback on individual aspects of the CMQ, such as re-ordering open and closed questions, and adapting the response options for the Child Report section. The final version of the CMQ is included in the supplementary material, and is freely available for anyone to use. We also developed a Good Practice Guide to support families and professionals when using the CMQ.

The aim of developing the CMQ was to meet the need for tools to help children, their families, and those who work with them, better understand their experiences of masking. Following feedback from the participants in these studies, we feel more confident that the CMQ can be used by adults to report on children’s masking strategies, and by some children to reflect on their own masking. However we are also aware of the limitations to a self-report questionnaire approach, as were raised by some of the participants in these studies. Ability to complete the Child Report section of the CMQ is limited by children’s abilities to self-reflect, and identify relatively abstract behaviours or experiences, and so this tool will not be suitable for all children. Some may require more support, for instance rephrasing questions or giving more personalised examples. Some children may only complete the open questions, and might choose to do so in more creative ways (such as drawing) which require interpretation by the person administering the CMQ. We acknowledge that the tool is not suitable for all children, but encourage families and professionals to consider the suitability on a case-by-case basis, with particular attention paid to whether the child would benefit from exploring their experiences of masking. Not all autistic or neurodivergent children mask (6, 23), and not all those who do mask may want or need to explore masking (24). As was noted by some participants in our studies, children who do not already mask might feel an expectation to start masking if the concept is introduced to them. However more research is needed to determine whether children such as these may still be experiencing negative consequences of having stigmatised identities (28), whether or not they mask as a result of this.

The inclusion of both adult and child perspectives on masking was welcomed by participants, who felt that both groups can provide useful insight on a child’s experiences of masking. Those completing the CMQ could compare answers between the adult and the child to identify masking strategies that may be more externally visible (and reported more by observers; 24), compared to those strategies which may not produce much visible change in behaviour (reported by the child only). This could also aid the identification of masking strategies that may require a lot of effort from the child, but may not result in the desired effect as perceived by an observer, encouraging reflection by the child on whether or not they want to continue using these strategies. The adult response section could also be completed by multiple different adults for the same child (e.g. a parent and a teacher), to allow comparison of masking across different environments (10, 16). This may be useful as part of developing a wider strategy to support the child across school, educational, and clinical settings.

One strength of this project was the range of families who were included in the evaluation, including children of different ages, and those with and without multiple different neurodivergent conditions. This reflects the target population for this tool: families of children who may be neurodivergent and may mask their neurodivergence. It was important to the Collective when developing this tool that it is suitable regardless of whether or not a child has a formal diagnosis of autism, as masking occurs in neurotypical children and in those who are awaiting formal assessment (23, 29). We note that the CMQ was developed to measure masking of autistic traits, but that non-autistic children may still have some autistic traits that they mask – the CMQ therefore should not be used to identify autism (12). In addition, we also emphasise that the CMQ is intended to be scored qualitatively, although it is possible to convert responses to the closed questions into numerical scores. As such, it is not appropriate to directly compare CMQ scores between groups or individuals, but instead a qualitative summary should be produced by combining closed and open question responses.

The CMQ also only measures certain aspects of masking, namely behavioural expressions consciously identified by the child themselves or observed by adults around them, and therefore should not be considered a comprehensive assessment of a child’s entire experience of masking (2, 3). It may not be possible to measure all aspects of masking (including but not limited to intent, strategies, effort, impact/effectiveness, immediate cost and longer term/mental health impact) in one approach, but other methods should be developed and evaluated to address these important aspects as well. We also acknowledge that in some cases masking may not be a conscious experience (28), and the CMQ does not capture aspects of masking which are unconscious or of which children are not (yet) aware 20, 21).

A limitation of our approach is that the CMQ has not been tested in a formally diagnosed sample, and we have not compared how the CMQ performs between autistic children, those with other neurodevelopmental conditions, and typically developing children who may also mask aspects of their identity, such as social anxiety (30). This is a common issue with tools developed to measure masking (3, 31), and requires addressing through large-scale evaluation in clinical and non-clinical samples. Responses on the CMQ could be compared with answers on established measures of impression management, stigma, and other conceptually related constructs, to provide evidence regarding the appropriateness of the CMQ for measuring masking of autistic traits versus other differences.

Another limitation is that we did not use a formal or structured analytical approach when evaluating feedback on the CMQ, due to the variation in data collection methods and timeframes between the two studies, meaning raw data could not be combined across studies. We recommend that future evaluations of the CMQ and/or other measures use more standardized approaches such as content analysis, to improve the rigour of analyses.

In conclusion, this paper introduces the Children’s Masking Questionnaire as one tool which could be used to measure masking in children aged 8 years and up. While it requires further rigorous evaluation, the CMQ appears to be a useful and appropriate tool to be completed by adults and by children themselves.

## Data Availability

The datasets presented in this article are not readily available due to the potentially identifiable nature of qualitative responses. Requests to access the datasets should be directed to the corresponding author.
