# Supplementary material for: Development and pilot evaluation of the Children’s Masking Questionnaire
Source: Front Psychiatry. 2026 Jul 23;17:1893936. doi: 10.3389/fpsyt.2026.1893936 (PMC13442543; doi:10.3389/fpsyt.2026.1893936)
Supplement: Supplementary file 1 [file DataSheet1.pdf]

Children's Masking Questionnaire: Adult Response

This questionnaire asks about behaviours that all children do to some extent, however some children may do more often than others.

Please think carefully about whether you have ever seen the child do any of these behaviours, or if they have told you about doing any. We are interested in whether children do these behaviours when they are around other people. If the child does any of these behaviours when they are by themselves, please note this in the comments.

The response should reflect how often the child has done this in the past month. If you can't recall exactly, please give your best estimate. Never = not at all; Rarely = one to four times in a month; Sometimes = once per week; Often = multiple times per week; Always = every day.
Please add examples of anything specific you can remember the child doing in the past month.

Around other people, does the child ever:	This might look like (but doesn't have to include):	Response: (never, rarely, sometimes, often, always)	More detail:
Suppress involuntary noises or actions such as hand flapping, tapping, touching objects	Not flapping their hands when excited		
	Stopping themselves from humming or singing when they are focused on something		
Feel like they can't talk about what they want to	Not sharing interests or talking about them less than they normally would		
Hide their emotions and preferences	Smiling when feeling upset or overwhelmed		
	Feeling pressure to always be 'ok'		
Let their emotions out at home	Becoming upset or angry after coming back from school		
	Needing quiet time alone after socialising		
Put on an act	Having a persona (quiet, chatty) to use in social situations		
	Pretending to be a character or someone else to get through social interactions		
	Making eye contact even if it's unpleasant		

Children's Masking Questionnaire © 2024 by Masking Research Collective: Laura Hull, Saffron Baldoza, Hannah Belcher, Georgia Ellin, Sarah O'Brien, Jo Halsall, & Lucy Sanctuary is licensed under CC BY-SA 4.0

Force themselves to make eye contact	Looking at someone's ears instead of in their eyes		
Change their voice or language used	Adjusting speech volume or tone to sound more like other children		
	Using a different vocabulary with other people than they would normally do		
Change their mannerisms to fit in with others	Using gestures more/less or differently than they would normally do		
Practice what to say	Rehearsing questions to ask when meeting new people		
	Checking whether certain things are ok to talk about		
Fit in with what is 'normal'	Trying to be like everyone else		
	Being worried about not fitting in		
Study or practice facial expressions	Looking at or copying facial expressions of characters in books or on TV		
	Practicing facial expressions in the mirror		
Suppress their sensory experiences	Not blocking their ears at distressing noises		
	Pretending they aren't upset by smells that they usually dislike		
Pretend to understand social interactions they don't normally understand	Smiling or laughing at jokes they don't get		
	Pretending to understand group dynamics, such as how bullying works		
Compensate for difficulties interacting with other children	Learning or asking about the rules of a game before joining in		
	Taking control of conversations with others		
Hide their support needs (if the child has no support needs, answer 'Never')	Not engaging with support staff at school		
	Not telling other children about any support needs they have		

Children's Masking Questionnaire © 2024 by Masking Research Collective: Laura Hull, Saffron Baldoza, Hannah Belcher, Georgia Ellin, Sarah O'Brien, Jo Halsall, & Lucy Sanctuary is licensed under CC BY-SA 4.0

Pretend to interact with other children	Playing near to other children but not with them		
	Sitting in group conversations but not contributing		
Copy other children	Mimicking other children's gestures or voices		
	Watching another child to learn how they act		
Act differently across different situations	Seeming more like 'themselves' at home than in other places		
	Having different versions of themselves for different situations		
Please add anything else we didn’t ask about that you think might be relevant.			

Children's Masking Questionnaire © 2024 by Masking Research Collective: Laura Hull, Saffron Baldoza, Hannah Belcher, Georgia Ellin, Sarah O'Brien, Jo Halsall, & Lucy Sanctuary is licensed under CC BY-SA 4.0
