# Supplementary material for: Development and pilot evaluation of the Children’s Masking Questionnaire
Source: Front Psychiatry. 2026 Jul 23;17:1893936. doi: 10.3389/fpsyt.2026.1893936 (PMC13442543; doi:10.3389/fpsyt.2026.1893936)
Supplement: Supplementary file 2 [file DataSheet2.pdf]

Children's Masking Questionnaire: Child Activity

This section can be completed by the child/young person on their own, or with an adult's support.
Please note here who completed this:
Child alone ☐ Child with adult's support ☐

This survey asks about some behaviours that all children might do. We want to know more about whether you do these things, and what you think about them. There are no right or wrong answers, we are interested in what you think. It's ok if you don't know how to answer.

Section 1.
Please answer these questions as best as you can. You can write, draw, or doodle your answers.

Do you ever feel like you need to hide or change things about yourself when you are around other people?

If yes, what things do you feel like you need to change? What do you do when you feel like this?

Section 2.

	Never	Sometimes	Always	At home	At school	More detail or in other places:
You can use these answers to show how often you do each behaviour, and where you do it. If you want to tell us more about your answer, you can put this in the 'more detail' section.	Not at all	A little bit	A lot	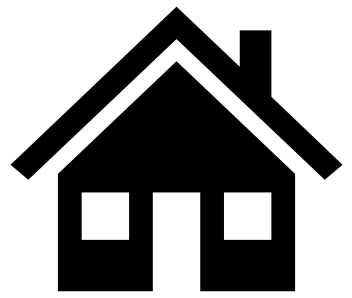	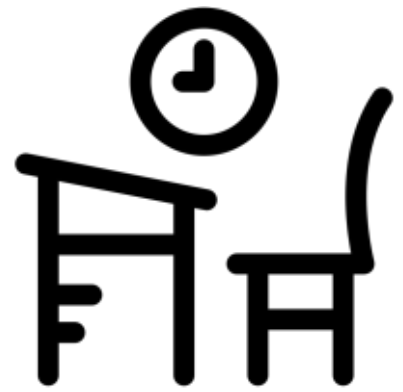	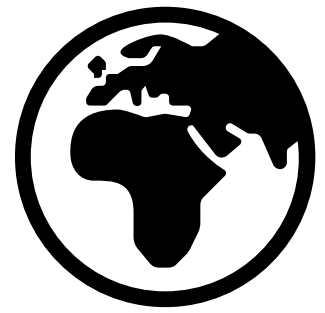
**Do you ever:**						
Stop yourself from making sounds or movements that you would normally make?						
Feel like you can't talk about things you like talking about?						
Hide how you are feeling or what you want to do?						
Feel tired or angry after being with other people?						
Pretend to be someone else when talking to other people?						

You can use these answers to show how often you do each behaviour, and where you do it. If you want to tell us more about your answer, you can put this in the ’more detail’ section.	Never	Sometimes	Always	At home	At school	More detail:
	Not at all	A little bit	A lot	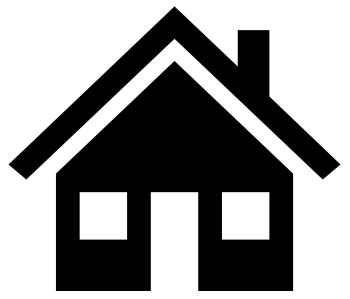	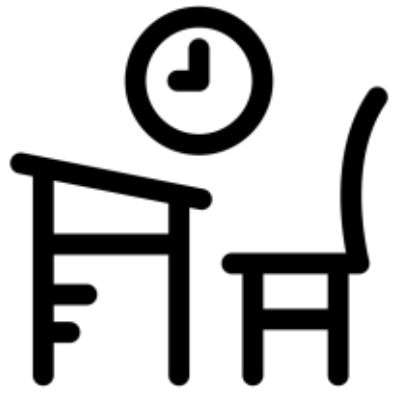	
Make eye contact (or pretend to) even if you don’t want to?						
Change the way you speak to fit in with other people?						
Practice things to say when talking to other people?						
Worry about not fitting in with other people?						
Look at or copy facial expressions, such as from TV characters?						

	Never	Sometimes	Always	At home	At school	More detail:
You can use these answers to show how often you do each behaviour, and where you do it. If you want to tell us more about your answer, you can put this in the ’more detail’ section.	Not at all	A little bit	A lot	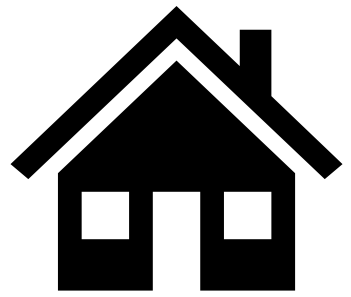	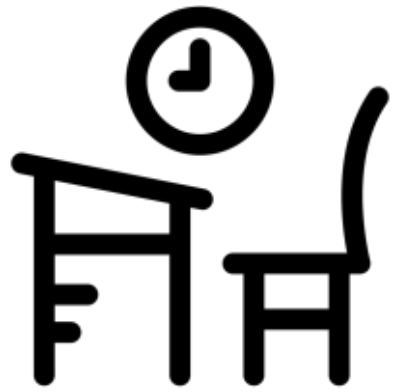	
Stop yourself from showing other people your reactions to sounds, smells, or lights?						
Pretend to laugh at jokes when you don’t understand them?						
Pretend that you understand why other children are acting in a certain way?						
Copy the way other children talk or act?						
Act like a different version of yourself with different people?						
**Please add anything else you do like this that we didn’t ask about:**						

CHILDREN'S MASKING QUESTIONNAIRE V1.0 (August 2024)

Children's Masking Questionnaire © 2024 by Masking Research Collective: Laura Hull, Saffron Baldoza, Hannah Belcher, Georgia Ellin, Sarah O'Brien, Jo Halsall, & Lucy Sanctuary is licensed under CC BY-SA 4.0
