# Supplementary material for: Development and pilot evaluation of the Children’s Masking Questionnaire
Source: Front Psychiatry. 2026 Jul 23;17:1893936. doi: 10.3389/fpsyt.2026.1893936 (PMC13442543; doi:10.3389/fpsyt.2026.1893936)
Supplement: Supplementary file 3 [file DataSheet3.pdf]

Children's Masking Questionnaire: Good Practice Guide

1. **How to complete the CMQ**
    a. Adult Report

The CMQ Adult Report section should be completed by an adult who knows the child well, i.e. has interacted with them across a variety of situations for a meaningful amount of time. This could include a parent/carer, other family member, education worker (e.g. teacher, teaching assistant, playground supervisor), or clinical professional (e.g. educational psychologist, clinician, therapist). If you are not sure whether you are familiar enough with the child, add a note to the CMQ stating how long you have known the child for and in what capacity.

The CMQ Adult Report Section contains a list of behaviours which you may or may not have observed in this child or other children. After each general behaviour type, one or more examples of this behaviour are given. If you do not recognise the examples in the child, but still think that they show behaviours that fit into the general behaviour type, please fill in the frequency question anyway. Please note down any other examples of this behaviour that you have seen this child do in the 'more detail' section. You can also use this space to share more information about the behaviour – such as situations in which they do this, or how they act before or after the behaviour. The 'more detail' section is a chance for you to share anything else related to the behaviour that you think might be important to consider.

    b. Child Activity

The Child Activity section has two parts: Section 1, where children can enter free responses to open questions, and Section 2, where children are asked to rate specific masking statements. It should be completed by children aged 8-16 years (including children aged above or below this depending on their reading and writing abilities), and can be completed with the support of an adult. If an adult does help the child to complete the Child Activity, please note this at the start.

Section 1. This section includes a few open questions where children are encouraged to answer freely. They can write, draw, doodle, or use other methods to answer. Feel free to use the back of the question sheet to continue answers if needed. The questions are designed to be open and don't refer explicitly to either masking or autism. We want to make sure that all children feel like they can complete this, regardless of whether or not they have heard of 'autism' or 'masking' before. Children may choose to answer based on other aspects of their identity besides neurodivergence; this is ok.

Section 2. This section includes some short examples of masking strategies described by other children. Children are asked how often they do each behaviour (not at all, a little bit, or a lot), and whether they do it at home and at school. They can tick or mark the appropriate box, and there is space for more detail if needed. More detail can be added within the question space if it does not fit in the more detail box. If this section is being completed with the support of an adult, feel free to explain or rephrase the question if the child is struggling to answer. There is also space at the end to add any additional examples of masking behaviours that haven't been identified so far.

2. **How to interpret the answers**
    a. Adult Report

There are two ways to interpret the answers to the CMQ questions. Firstly, you can read all of the answers together and use these to produce a summary of the child's potential masking

Children's Masking Questionnaire: Good Practice Guide © 2024 by Masking Research Collective: Laura Hull, Saffron Baldoza, Hannah Belcher, Georgia Ellin, Sarah O'Brien, Jo Halsall, & Lucy Sanctuary is licensed under CC BY-SA 4.0

behaviours. For instance, which behaviours were reported as occurring more frequently? These could be highlighted as key masking behaviours shown by this child. Overall you should be able to produce a short summary of ways in which the child masks, which can be shared with other people.

The second option is to assign a numerical score to each answer. Convert each answer into its corresponding number. Never = 1, Rarely = 2, Sometimes = 3, Often = 4, Always = 5. Add up all the scores, and you should have a number between 18 and 90. Higher scores mean greater masking, however there is no 'cut-off' or threshold above which a child can be considered to be masking. This is because masking is an individual behaviour and its impact will be different for each child. Producing a numerical score can be useful for comparing between large numbers of children, however for the purposes of supporting an individual child, it may be more helpful to use the 'summative' approach described above.

b. Child Activity

The answers to Section 1 can be used as the start of a broader discussion about masking, if this is helpful. If the child drew or used pictures to answer, you might want to ask them to explain more about the picture.

Section 2 can be scored numerically where not at all = 0; a little bit = 1; and a lot = 2. You can then add the total score together to get a sense of how much the child masks overall, or look at individual items where the child has scored 2 to identify specific masking behaviours they may use a lot. The school/home response will also help you think about where the child masks most of the time, as well as who should be aware of this.

3. What to do with the answers

a. Advice for families

Once you have produced a summary of all the answers, reflect on this. Does it fit with your understanding of how the child acts in different situations? Or were there any answers that surprised you? Consider starting a conversation with the child about their masking. Some questions you could include, making reference to specific behaviours as appropriate:

- How do you feel when you do this? How do you feel afterwards?
- Have you ever tried doing something different? How did that go?

It may also be useful to talk with the child, and with other family members, about situations where they feel like they don't need to mask. When can they be completely themselves, and what do they do? What's different about those situations (e.g. who else is there, the sensory environment)? Would they like to have more of those situations? This could help you be more aware of when the child might feel like they need to mask, and so keep an eye on their wellbeing and energy levels at that time.

Research suggests that masking is associated with poor mental health outcomes for autistic adults, although there is still little evidence regarding children. We also know that for some children, masking can lead to positive outcomes such as making friends and feeling accepted, therefore it's important to consider exactly what masking means for each individual child, before deciding whether it is a 'good' or 'bad' thing.

Children's Masking Questionnaire: Good Practice Guide © 2024 by Masking Research Collective: Laura Hull, Saffron Baldoza, Hannah Belcher, Georgia Ellin, Sarah O'Brien, Jo Halsall, & Lucy Sanctuary is licensed under CC BY-SA 4.0

Some resources which might help you when having these conversations:

- Autism and Masking (Sedgewick, Hull & Ellis) – this book (which was co-written by one of the developers of the CMQ) offers advice on how to support autistic people who mask. It is mostly aimed at adults, but there may be some useful insights to apply to children.
- Know Your Normal (Ambitious about Autism) – a resource co-developed with autistic young people to help understand your mental health and wellbeing, to get a sense of what is 'normal' for you and how to identify when things change from normal.

b. Advice for educational professionals

Once you have produced a summary of all the answers, reflect on this. Does it fit with your understanding of how the child acts in different situations? Or were there any answers that surprised you? Consider starting a conversation with the child about their masking. Some questions you could include, making reference to specific behaviours as appropriate:

- How do you feel when you do this? How do you feel afterwards?
- Have you ever tried doing something different? How did that go?
- What can we do to help you feel more like yourself at school? [make suggestions of specific changes, such as being able to wear ear defenders/fiddle with toys, which could be added to the child's EHCP if appropriate]

It may also be useful to talk with the child, and with other staff members, about situations where they feel like they don't need to mask at school or at home. When can they be completely themselves, and what do they do? What's different about those situations (e.g. who else is there, the sensory environment)? Would they like to have more of those situations?

You can then think about making changes to the school environment to better support the child. For instance, this could include identifying a 'safe space' where the child can go to stop masking, have some time alone, and engage in sensory regulation. This may limit the negative impact that masking has. Another option could be to encourage greater awareness and acceptance of neurodiversity in the school, so that the child feels less expectation to mask if they don't want to. Resources to support this are listed below:

- LEANS – Learning about Neurodiversity in Schools. A free teacher-led programme to teach the whole class about neurodiversity. Currently for primary schools only.
- Training for school staff - children may feel expectation to mask outside of the classroom, for instance in dining spaces, playgrounds, or school trips. Training for all staff members, regardless of the how often they engage with a specific child, may create a more welcoming environment for neurodivergent children and so reduce expectations to mask.

c. Advice for healthcare professionals

Children's Masking Questionnaire: Good Practice Guide © 2024 by Masking Research Collective: Laura Hull, Saffron Baldoza, Hannah Belcher, Georgia Ellin, Sarah O'Brien, Jo Halsall, & Lucy Sanctuary is licensed under CC BY-SA 4.0

Once you have produced a summary of all the answers, reflect on this. Does it fit with your understanding of how the child acts in different situations? Or were there any answers that surprised you? Consider starting a conversation with the child about their masking. Some questions you could include, making reference to specific behaviours as appropriate:

- How do you feel when you do this? How do you feel afterwards?
- Have you ever tried doing something different? How did that go?
- What do you want people to know about you when you are doing this?

In healthcare settings it is important to be aware of masking, as it may result in a child presenting differently to the way you expect – for instance, hiding pain, discomfort, or distress. By identifying the ways in which a specific child masks, as well as what they do when masking, you can begin to identify times in which they may need support to stop masking, or where additional information may be helpful to understand a child's underlying experiences.

One option could be to share the summary of masking behaviours with other healthcare professionals who engage with the child, along with a list of strategies to support them to self-regulate, and potentially, to stop masking. This should be produced in collaboration with the child and their family.
Within therapeutic settings, the masking behaviours identified here could form targets for behavioural change or intervention, if considered appropriate by clinicians, the child, and their family. Alternatively, if masking behaviours are considered adaptive by the child and their family, with minimal negative consequences, this should be noted and potential 'coping strategies' could be expanded upon.

Resources to support this are listed below:

- Autism and Masking (Sedgewick, Hull & Ellis) – this book (which was co-written by one of the developers of the CMQ) offers advice on how to support autistic people who mask. It is mostly aimed at adults, but there may be some useful insights to apply to children.

Children's Masking Questionnaire: Good Practice Guide © 2024 by Masking Research Collective: Laura Hull, Saffron Baldoza, Hannah Belcher, Georgia Ellin, Sarah O'Brien, Jo Halsall, & Lucy Sanctuary is licensed under CC BY-SA 4.0
