# Supplementary material for: Development and pilot evaluation of the Children’s Masking Questionnaire
Source: Front Psychiatry. 2026 Jul 23;17:1893936. doi: 10.3389/fpsyt.2026.1893936 (PMC13442543; doi:10.3389/fpsyt.2026.1893936)
Supplement: Supplementary file 4 [file Table1.docx]

Supplementary material 1

**Survey questions for Study 1**

Qualitative Feedback on the Adult Response Section:

1. Were there any questions in particular that you found difficult to answer? Please list them here (you don't have to give the exact wording) and tell us why.

2. Any other feedback you would like to give about this section?

Qualitative Feedback on the Child Response Section:

1. What did you and/or the child think about the bricks slider as a way to answer the questions?

2. Did you change or rephrase any of the questions to help the child understand them? Please give examples if you can remember any.

3. Were there any questions that the child found particularly difficult to answer? Please list them here (you don't have to give the exact wording) and tell us why.

4. Any other feedback about this section?

5. If you did not complete this section, why was this? (e.g. you did not think the child would be able to answer; you thought it would take too long; the child was not around to complete it with you)
