# Supplementary material for: Development and pilot evaluation of the Children’s Masking Questionnaire
Source: Front Psychiatry. 2026 Jul 23;17:1893936. doi: 10.3389/fpsyt.2026.1893936 (PMC13442543; doi:10.3389/fpsyt.2026.1893936)
Supplement: Supplementary file 5 [file Table2.docx]

Supplementary material 2

**Cognitive Interview Schedule**

(note these questions may be adapted or added to during the interview)

Please read through the questionnaire and speak aloud your thoughts for each question. Here are some prompts to get you started:

1. Does the question make sense to you? Are there any words you don’t understand?

2. Do you have a ‘gut instinct’ of how you would answer this question?

3. Do you recognise the behaviour/action that is being described here (even if the child you are answering about does not do this)?

4. Would you ask this question in a different way?

Please answer these questions, reflecting on the questionnaire as a whole:

5. Do the answer options make sense? Would you like to answer in a different way?

6. Was there any information you thought was relevant that we didn’t ask for?

7. Was there any information we asked for that you thought was inappropriate, or you didn’t want to answer?

8. Do you think a child aged 8-9 years would be able to complete the Child Report section (with/without adult support)?

9. Do you think a young person aged 10-12 years would be able to complete the Child Report section (with/without adult support)?
