# Supplementary material for: Development and pilot evaluation of the Children’s Masking Questionnaire
Source: Front Psychiatry. 2026 Jul 23;17:1893936. doi: 10.3389/fpsyt.2026.1893936 (PMC13442543; doi:10.3389/fpsyt.2026.1893936)
Supplement: Supplementary file 6 [file Table3.docx]

Supplementary material 3

**Survey questions for Study 2**

(Note that only qualitative responses to these questions were analysed in the present study. Some questions received no qualitative answers, but we have included all questions for transparency).

For clinicians:

1. How clear did you find the questions in the CMQ?

2. How long did you find the CMQ?

3. How would you rate the accessibility of the CMQ?

4. How well did you understand the questions in the CMQ?

5. Was there anything in the CMQ that you did not understand or wish to be clarified?

6. How likely do you think your patients are to identify with any of the behaviours mentioned in the CMQ?

7. Are there any challenges you think you would face if you completed the CMQ?

8. Is there anything that would make it easier to complete the CMQ?

9. Do you think a child aged 8 or older could complete the Child Response section by themselves?

10. How relevant do you think the CMQ would be to your patients’ care?

11. How useful do you think the CMQ would be in identifying children who may be masking neurodivergence?

12. If you would use the CMQ in your practice, during which part of the assessment and management of your patients would you expect to use it?

13. To what extent do you think implementing the CMQ in [this service] would significantly impact your current workload?

14. Do you think there is anything missing from the CMQ?

15. Do you have any other feedback about your experience of completing the CMQ?

For families:

1. How clear did you find the questions in the CMQ?

2. How long did you find the CMQ?

3. How would you rate the accessibility of the CMQ?

4. How well did you understand the questions in the CMQ?

5. Was there anything in the CMQ that you did not understand or wish to be clarified?

6. Did you identify with any of the behaviours mentioned in the CMQ?

7. Are there any challenges you think you would face if you completed the CMQ?

8. Is there anything that would make it easier to complete the CMQ?

9. Would you be able to complete the Child Response section by yourself?

10. How relevant do you think the CMQ would be to your care?

11. Do you think there is anything missing from the CMQ?

12. Do you have any other feedback about your experience of completing the CMQ?
